# Severe Inbreeding and Small Effective Number of Breeders in a Formerly Abundant Marine Fish

**DOI:** 10.1371/journal.pone.0066126

**Published:** 2013-06-07

**Authors:** Shannon J. O'Leary, Lyndie A. Hice, Kevin A. Feldheim, Michael G. Frisk, Anne E. McElroy, Mark D. Fast, Demian D. Chapman

**Affiliations:** 1 School of Marine and Atmospheric Science, Stony Brook University, Stony Brook, New York, United States of America; 2 Pritzker Laboratory for Molecular Systematics and Evolution, The Field Museum, Chicago, Illinois, United States of America; 3 Atlantic Veterinary College, University of Prince Edward Island, Charlottetown, Canada; 4 Institute of Ocean Conservation Science, Stony Brook, New York, United States of America; Macquarie University, Australia

## Abstract

In contrast to freshwater fish it is presumed that marine fish are unlikely to spawn with close relatives due to the dilution effect of large breeding populations and their propensity for movement and reproductive mixing. Inbreeding is therefore not typically a focal concern of marine fish management. We measured the effective number of breeders in 6 New York estuaries for winter flounder (*Pseudopleuronectes americanus*), a formerly abundant fish, using 11 microsatellite markers (6–56 alleles per locus). The effective number of breeders for 1–2 years was remarkably small, with point estimates ranging from 65–289 individuals. Excess homozygosity was detected at 10 loci in all bays (F_IS_ = 0.169–0.283) and individuals exhibited high average internal relatedness (IR; mean = 0.226). These both indicate that inbreeding is very common in all bays, after testing for and ruling out alternative explanations such as technical and sampling artifacts. This study demonstrates that even historically common marine fish can be prone to inbreeding, a factor that should be considered in fisheries management and conservation plans.

## Introduction

McNeely et al. [1] defined three levels of biodiversity: ecosystem diversity, species diversity and genetic diversity. While ecosystem diversity describes the differences of habitats and environmental parameters that shape communities, species diversity describes the variety and abundance of organisms inhabiting a certain area and genetic diversity focuses on the combination and variation of genes found within a single population of one species. The conservation of genetic diversity is often not well incorporated into marine fish management [2] despite being the most fundamental level of biodiversity and a key source of variation that enables evolutionary adaptation [3], [4]. This stems from the fact that the key processes that reduce genetic diversity, such as inbreeding and stochastic gentic drift, are associated with very small, fragmented populations and are assumed to be diluted in large, well-mixed populations [5], [6], [7]. Since marine fish are traditionally assumed to exist as large, panmictic populations connected by larval and adult-mediated dispersal [8], [9], [10] it is not suprising that conservation of genetic diversity is not emphasized in marine fish conservation [2], [11].

Recent studies have shown that marine fish populations can be more structured than traditionally thought [12], [13], [14] and effective population size, which determines how vulnerable populations are to losing genetic diversity due to genetic drift, can be up to five orders of magnitude smaller than census population sizes in broadcast spawning species that exhibit large variance in reproductive success [12], [16], [17]. These findings have initiated a paradigm shift that marine fish may be more vulnerable to processes that reduce genetic diversity than previously assumed [17], [18], for example through inbreeding, defined in population genetics as a departure from random mating. Hoarau et al. [17] detected heterozygote deficiencies in plaice (*Pleuronectes platessa*) in the North Sea and, after ruling out alternative hypotheses, concluded that inbreeding was responsible for this pattern. Despite having a relatively large census population size, plaice tend to spawn in their natal area and have high variance in reproductive success, increasing the probability that spawning pairs or groups will contain related individuals. Despite this remarkable finding, there have been few follow-up studies of inbreeding in marine fish, even though heterozygote deficiencies have been detected in many other species, including redfin culter (*Culter erythropterus*) [14], anchovy (*Engraulis encrasicolus*) [15], rockfish (*Sebastes melanops*) [19] and whitefish (*Coregonus lavaretus*) [20]. It is therefore difficult to determine whether inbreeding in plaice is an anomaly or a process that should be of broader conservation concern for heavily exploited marine fish.

The winter flounder (*Pseudopleuronectes americanus*) is a demersal flatfish that was once very common in the inshore bays and estuaries of the Northwest Atlantic and supported very large commercial and recreational fisheries [21], [22]. The species' geographic range encompasses the North American coast from Labrador to Georgia [23]. Spawning migrations occur in the winter and spring months and there are four broadly defined and genetically discrete spawning stocks across the species range: Sable Island Bank, St. Mary's Bank, Browns Bank, and Georges Bank [24]. Winter flounder eggs are demersal and it has been assumed that pre-settlement larvae are mixed through larval dispersal within each stock [25]. However, more recent studies have shown that larvae are likely retained within their natal estuary [26] and a number of authors believe that adults remain within or return to their natal estuaries to spawn [27]–[31], both of which could promote the development of fine-scale population structure.

Winter flounder began declining in the late 1980s and the age structure of many populations has become truncated, with a low proportion of the remaining fish older than 5 years [22]. Long Island, New York (LI) is a very densely populated region with 2,086 people per km^2^, and has a long history of commercial and recreational exploitation of winter flounder [22]. Commercial [32], [33] and recreational [33] (http://www.st.nmfs.noaa.gov/recreational-fisheries/access-data/run-a-data-query/index) landings have reached record low levels, and despite management there is little evidence of recovery [22], [26], [34], [35]. In light of the extent of inbreeding observed in North Sea plaice, a fish with many life-history similarities to winter flounder, we tested for inbreeding in winter flounder in LI estuaries. We also tested for genetic differentiation among bays, estimated the effective number of breeders for each bay and tested for genetic bottlenecks, all of which contribute to the rate at which population genetic diversity is lost.

## Methods

### Ethics statement

Necessary permits for sampling and handling fish were obtained from responsible agencies and authorities (NY DEC Permit #1030 and 1644 for all bays, Gateway NRA (National Park Service) Permit #GATE-2007-SCI-0021 and GATE-2011-SCI-0014 for Jamaica Bay and a permission from the Town of East Hampton Trustees for Napeague Harbor). All winter flounder were sacrificed by being flash frozen on dry ice in the field as approved in our IACUC protocol (IACUC “Restoring Long Island's Winter Flounder Fishery”, IRBNet#: 260837-3).

### Sample collection

**Figure 1:**
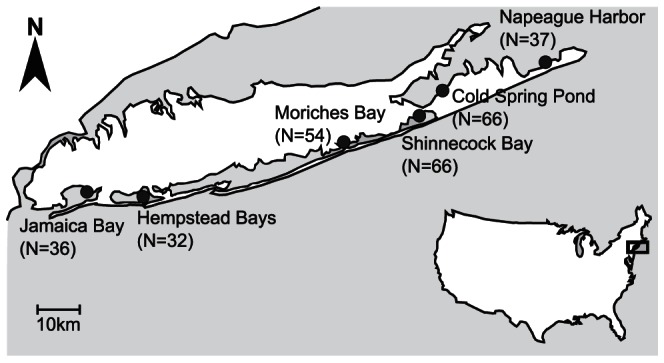
Sample locations and sample number (N) for 2010–2011.

Young-of-the-year (YOY) winter flounder were collected with a 1 m beam trawl every two weeks from June to October in 2010 and May to October in 2011. Samples were collected in 6 bays (Figure 1): Jamaica Bay (40 38′ 28.43″ N, 73 49′ 02.37″ W) in 2010 and 2011, Moriches (40 47′ 02.47″N, 72 47′ 23.14″W) in 2010 and 2011, Hempstead (40 36′ 58.85″N, 73 35′ 52.81″ W) in 2011, Shinnecock Bay (40 51′ 46.13″N, 72 29′ 44.73″W) in 2010 and 2011, Cold Spring Pond (40 53′ 59.04″N, 72 27′ 40.31″W) in 2010, and Napeague Harbor (41 00′ 34.62″N, 72 02′ 49.84″W) in 2010. With the exception of Hempstead Bays, trawls within each bay occurred at 10 randomly chosen stations within a grid along a section of coast where winter flounder had previously been caught. Supplementary sampling occurred in 2011 throughout Hempstead Bay using a beam trawl or 3–30 m beach seines due to low abundances of fish in this area. Fin clips were taken from all flounder and stored in 75% reagent grade ethanol at room temperature.

### Laboratory analysis

Genomic DNA was extracted from fin clips (0.015–0.035 mg of tissue) using the Qiagen Blood and Tissue extraction kit (Qiagen, Valencia, CA, USA). Eleven microsatellite loci were amplified using PCR primers that were either used for previous winter flounder microsatellite studies (five loci [24], [36]) or recently developed (six loci [37]). The master mix consisted of 1× PCR buffer, 10× bovine serum albumin, 1.5–3.5 mM MgCl_2_, 0.12 mM of each dNTPs, 0.16 µM of the reverse primer and the fluorescently labeled M13 primer and 0.04 µM of the species specific forward primer and 1 unit Taq polymerase in a final reaction volume of 10 µl. Thermal cycling profiles were 4 minutes at 94°C, 30 cycles of 94°C for 15 seconds, primer specific annealing temperature for 15 seconds, 72°C for 45 seconds and 5 cycles of 94°C, 15 seconds at 53°C and 45 seconds at 72°C with a final extension for 10 minutes at 72°C. Locus-specific annealing temperatures (T_a_) are given in Table 1. Amplified products were separated and sized using an internal size standard (LIZ-500 Applied Biosystems) on a Genetic Analyzer (Applied Biosystems ABI3730 sequencer). Alleles were scored by a single analyst (SO) using Peakscanv1.0 (Applied Biosystems). For quality control, a second analyst (KAF) verified the scoring of approximately 30% of heterozygotes and 100% of the homozygotes.

**Table 1:** Genetic diversity for each microsatellite locus over all sample locations described through allelic richness and heterozygosity.

Locus	T_a_	K	H_o_	H_e_
A441 [38]	52	17	0.688	0.862
J42 [38]	52	19	0.764	0.844
Pam21 [26]	46	16	0.677	0.787
Pam27 [26]	47	20	0.638	0.869
Pam79 [26]	43	38	0.564	0.956
WF06 [39]	45	9	0.561	0.520
WF12 [39]	45	6	0.591	0.662
WF16 [39]	54	26	0.616	0.803
WF27 [39]	56	56	0.758	0.963
WF32 [39]	54	20	0.364	0.758
WF33 [39]	56	48	0.689	0.954

T_a_: Annealing temperatures. K: number of alleles. H_e_: expected heterozygosity. H_o_: observed heterozygosity.

### Testing for technical artifacts

The entire genotypic database was analyzed with Microsatellite Toolkit for Excel and Microchecker [38] to check for possible scoring errors, identical genotypes, large allelic dropouts, null alleles and large allelic gaps. Null alleles occur when there is a mutation within the binding site of the primer, causing annealing failure during PCR. If null alleles exist in the population, some individuals will be homozygous for these alleles at a certain locus and will consistently fail to amplify at this locus despite working at others. If null alleles were occurring at high frequencies we would therefore expect chronic issues with gaps within the dataset. To assess this possibility we attempted to re-amplify any samples that failed on the first attempt, making a dedicated effort to obtain a genotype at every locus for all individuals. When null alleles are present a proportion of individuals scored as homozygotes are actually heterozygotes for a null allele. We attempted to reveal these “false” homozygotes by reducing the stringency of the PCR. Following Hoarau et al. [17] a subset of 8 homozygotes for each locus was re-amplified at lower temperatures (3°C lower than the T_a_ listed for each locus in Table 1) to promote the amplification of null alleles, i.e. if null alleles were present we would expect additional alleles to be amplified in an individual previously scored as homozygotes thus revealing a proportion of homozygotes to be false.

### Statistical analysis

Expected and observed heterozygosity [39] and allelic richness were calculated for each sampled estuary using Fstat [40]. Genepop [41], [42] was used to test for linkage disequilibrium and deviations from Hardy-Weinberg Equilibrium (HWE) using exact tests. We calculated global F_ST_ to estimate population differentiation and pairwise F_ST_ to assess genetic differentiation between bays and tested for significance as implemented in Fstat [40]. The shortest distance by sea between each pair of sample locations was measured using Google Earth V.6.2.2.6613 to assess isolation by distance (IBD). The relationship between genetic similarity (M = (1/F_ST_)−1)/4 [43] and geographic distance was evaluated using Mantel tests [44] as executed in IBDWS [45].

Deviations from HWE can arise from inadvertent grouping of multiple populations into one or from analyzing a large number of related individuals (Wahlund effect). To test for the possibility of genetically distinct groups of winter flounder spawning in the same bay at different times we calculated pairwise F_ST_ for several temporal groups. We first tested all samples caught in 2010 (N = 115) against those caught in 2011 (N = 99). We then pooled samples caught in the early sampling season (May–July) and the late sampling season (August–October) and calculated pairwise F_ST_ for the following four temporal groups: early 2010 (N = 89), late 2010 (N = 26), early 2011 (N = 70) and late 2011 (N = 29). The program Structure [46] was also used to estimate the number of distinct genetic populations in LI by using a Bayesian approach to detect clusters of individuals that would minimize disruptions from HWE within the whole sample set. Structure was run using the admixture and the non-admixture model both using and not using a priori information regarding the sampling location. We simulated K = 2–15 for 10 independent runs each to determine convergence with a burn-in period of 15,000 Markov chain Monte Carlo (MCMC) steps followed by 350,000 MCMC steps. This approach is capable of detecting if there were strongly differentiated groups (F_ST_>0.05 [47] spawning in the same bays at different times that were admixed in the sample we collected. Lastly, we tested the pairwise relatedness of all individuals and calculated the average within group relatedness of individuals at each sample location in order to detect family structure within each sample.

The effective number of breeders (N_b_) was estimated for each bay and LI using the linkage disequilibrium method as implemented in LDNe [48], [49] with the lowest included allele frequency p_crit_ = 0.02. This method estimates the effective number of breeders based on a single sample by using the small level of linkage of alleles that occurs due to sampling error during recombination. Since we used young-of-the year individuals sampled over 1–2 years, the parameter we estimated is the number of breeders that effectively produced the sampled cohorts, not the effective population size Ne for the whole generation [50]. Three tests for genetic bottlenecks were implemented for LI as a whole and for each bay: the m-ratio test [51], the mode shift test [52] and the heterozygote excess test. The last two methods are implemented in the program Bottleneck [53].

Three metrics were used to estimate levels of inbreeding. At the sample (i.e., bay) level, the inbreeding coefficient F_IS_ [54] was used to measure the intrapopulation heterozygosity deficiency resulting from inbreeding when alleles are shared by descent. At the individual fish level, the internal relatedness (IR) was used to measure the relatedness of individual's parents [55]. For outbred individuals IR should be close to or below zero, whereas individuals derived from consanguineous mating have an IR that is positive (to a maximum of 1), indicating that the parents of that individual shared many alleles and are closely related. We used the program Storm [56] to calculate IR for each individual. We tested for significant difference between the mean IR levels of each bay using a t-test to determine significant differences between sample locations. In addition we calculated the homozygosity levels of individuals using Storm [56], which indicates the proportion of loci within the genotype of an individual that are homozygous.

## Results

Eleven microsatellite loci were amplified in 267 individuals sampled in 6 LI bays (32–66 individuals per bay; Figure 1). Loci had from 6 to 56 alleles (mean = 25, s.d. = 15.8) and observed heterozygosities ranging from 0.364 to 0.764 (Table 1). Microchecker did not detect evidence of stuttering or large allelelic drop out at any locus. While Microchecker did suggest the presence of null alleles due to strong deviations from HWE, none of the homozygotes exhibited an additional allele after being re-amplified at lower annealing temperatures (N = 8 homozygotes per locus). All individuals were successfully amplified and genotyped at all loci, indicating that there were no individuals that were homozygous for null alleles (i.e., there were no blanks in the dataset).

**Figure 2:**
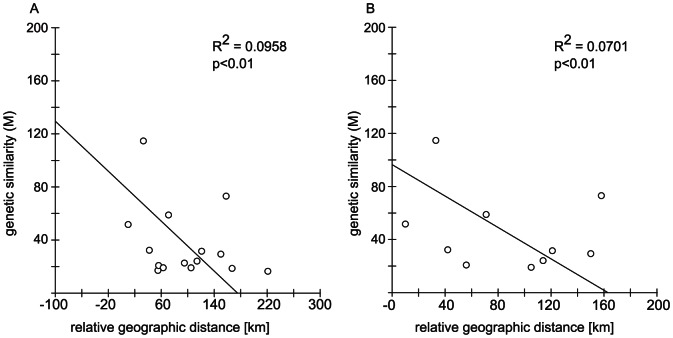
Isolation by distance. (2a) Regression of pairwise geographic distance and genetic similarity of all sample populations. (2b) Regression of pairwise geographic distance and genetic similarity of all sample locations excluding Napeague.

The global F_ST_ was small but significantly different from zero (F_ST_ = 0.008; p<0.05). Pairwise F_ST_ values between bays were consistently significant (p<0.05) for Napeague Harbor (except compared to Hempstead Bay) and Shinnecock Bay (Table 2), though they were no longer significant after Bonferroni correction for multiple comparisons. Analysis of IBD (Figure 2) was also significant, though weak (r^2^ = 0.0958, p<0.01). Even after removing Napeague Harbor, the most divergent and distant site, the IBD pattern was still significant (r^2^ = 0.0701, p<0.01). Temporal pairwise F_ST_ calculated between the years 2010 and 2011 was 0.005 (p = 0.4); pairwise F_ST_-values for comparison between the early and late sampling season ranged between 0.005 and 0.010 (p = 0.3–0.6). Bayesian clustering implemented in Structure failed to detect population structure from K = 2–15 for the entire sample set. Additionally, analysis of pairwise relatedness of all sampled individuals showed that less than 6% were related at a level of half-siblings or higher (r>0.25).

**Table 2:** Pairwise F_ST_ for all sample locations.

	Cold Spring	Jamaica	Hempstead	Moriches	Napeague	Shinnecock
**Cold Spring**		0.0104	0.004*	0.008	0.014**	0.005
**Jamaica**	0.187		0.012	0.005	0.013	0.013*
**Hempstead**	0.023	0.120		0.008	0.015*	0.009*
**Moriches**	0.093	0.280	0.150		0.011**	0.002**
**Napeague**	0.003	0.060	0.023	0.010		0.013**
**Shinnecock**	0.387	0.027	0.037	0.010	0.007	

F_ST_ values are given above the diagonal and p-values are presented below the. Significant values before Bonferroni correction are indicated. Indicative adjusted nominal level (5%) for multiple comparisons is: 0.0033.

* (p<0.05).

** (p<0.01).

**Figure 3:**
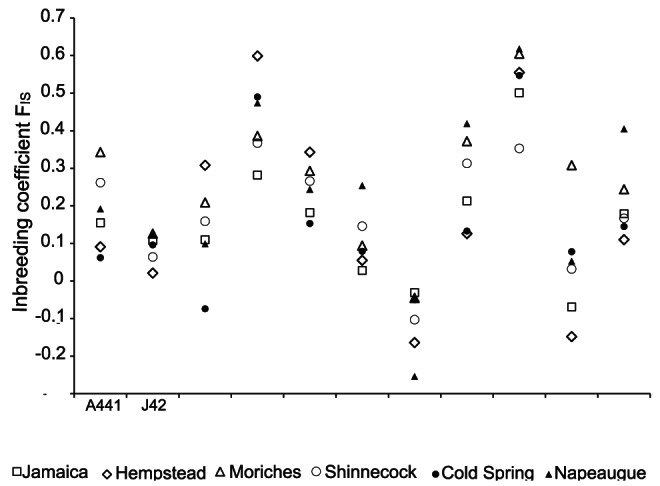
Inbreeding coefficient F_IS_ for all sample locations per microsatellite locus.

**Figure 4:**
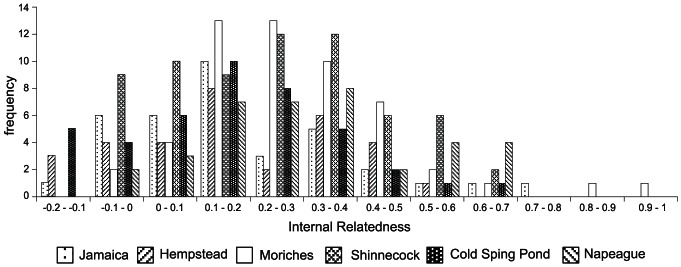
Internal relatedness. Frequency distribution of level of internal relatedness (IR) over all loci for all sample locations.

Overall genetic diversity as measured by heterozygosity and allelic richness was similar among all bays (Table 3). The estimated effective number of breeders for the sampled bays and LI overall were low (Table 4) ranging from 65–262 breeding individuals per bay and 966 overall. The L-mode shift test exhibited a non-bottlenecked distribution of alleles and the heterozygote excess test for bottlenecks was not significant (p<0.98). The m-ratios for the LI collection as a whole were generally high, whereas all loci but 3 (A441, J42 and WF12) exhibited moderate to low m-ratios within individual bays when using the entire range (R) of alleles found in all LI locations (Table 5). Large, significant discrepancies between expected and observed heterozygosities were detected at all loci in all bays (Table 1). Global Hardy-Weinberg testing of heterozygote deficiency were statistically significant at all bays (p<0.001 for all sample locations) and all loci (p = 0.00–0.0043 except WF06, p = 0.7). F_IS_ values over all loci were significantly different from zero and positive in all 6 bays, ranging from 0.169–0.283 (Figure 3). All loci except WF6 exhibited this pattern. The average in-group relatedness r per bay ranged r = −0.052–0.004. 94% of all values of pairwise relatedness were r<0.2. Average internal relatedness of individual fish was high, though highly variable (mean 0.229, s.d. 0.206) with a range of IR from −0.178–0.999 (Figure 4). This pattern was common to all 6 bays (Figure 4). In addition, pairwise comparison of mean IR of sample location shows some significant differences (Table 6). Moriches and Napeague have significantly higher mean IR values than Cold Spring Pond, Hempstead and Jamaica Bay (p<0.05), while Shinnecock has a significantly higher mean IR value than Cold Spring Pond. The internal level of homozygosity (HL), i.e. the proportion of loci of an individuals' genotype that were homozygotes, ranged from HL = 0–1. The mean value of the internal homozygosity level of individuals as highest for Morriches and Napeague with HL = 0.414 (std = 0.172) and HL = 0.427 (std = 0.172), respectively, and lowest for Cold Spring Pond and Hempstead Bays (HL = 0.296, std = 0.168 and HL = 0.314, std = 0.166) indicating that on average 30–40% of an individuals' loci are homozygous.

**Table 3:** Multi-locus genetic diversity for each sampling location (sample size N) and overall.

Location	N	H_e_	H_o_	A
Cold Spring	42	0.8255	0.701	16.18
Hempstead	32	0.8264	0.656	14.09
Jamaica	36	0.8248	0.6763	13.91
Moriches	54	0.7995	0.5847	16.73
Napeague	37	0.8013	0.5818	13.45
Shinnecock	66	0.7968	0.6214	17.27
LI	267	0.8174	0.6334	25.09

H_e_ expected heterozygosity over all loci. H_o_: observed heterozygosity over all loci. A: Allelic Richness (mean number of alleles per locus adjusted for sample size).

**Table 4:** Estimated effective number of breeders (N_b_) and 95% CI for all sample locations.

Location	N_b_	95% CI
Cold Spring	141.4	111.6, 190.6
Hempstead	113.7	83.9, 173.3
Jamaica	65.3	25.3, 166.3
Moriches	262.5	189.0, 421.2
Napeague	88.0	71.6, 112.8
Shinnecock	289.7	219.9, 418.6
LI Bays	966.1	808.1, 1195.0

**Table 5:** M-ratio calculated for all loci and sample location and overall sample locations.

	A441	J42	Pam21	Pam79	Pam27	WF12	WF6	WF33	WF32	WF16	WF27
Jamaica	0.47	0.89	0.33	0.67	0.41	0.5	0.58	0.46	0.2	0.39	0.23
Hempstead	0.71	0.74	0.33	0.29	0.41	0.83	0.66	0.43	0.29	0.47	0.37
Moriches	0.76	0.89	0.61	0.62	0.67	0.5	0.5	0.46	0.27	0.42	0.37
Shinnecock	0.65	0.84	0.61	0.57	0.52	0.83	0.67	0.46	0.34	0.36	0.5
Cold Spr	0.65	0.95	0.5	0.52	0.52	0.83	0.67	0.42	0.22	0.44	0.45
Napeague	0.76	0.68	0.44	0.45	0.52	0.83	0.5	0.34	0.2	0.36	0.33
overall	1	1	1	0.83	0.81	1	0.75	0.74	0.46	0.72	0.7

**Table 6:** Pairwise comparison of mean Internal Relatedness (IR) values per bay.

Average IR	Cold Spring	Hempstead	Jamaica	Morriches	Napeque	Shinnecock
**Cold Spring**	**0.1532**	0.5588	0.4461	0.0011	0.0015	0.0351
**Hempstead**	NS	**0.1801**	0.8488	0.0176	0.0164	0.2082
**Jamaica**	NS	NS	**0.1900**	0.0393	0.0340	0.3293
**Morriches**	**	*	*	**0.2883**	0.7956	0.1388
**Napeague**	**	*	*	NS	**0.2995**	0.1169
**Shinnecock**	*	NS	NS	NS	NS	**0.2342**

Bolded values on the diagonal are the mean IR values of individuals sampled at the designated location, p-values of pairwise t-test are shown above the diagonal and significance level before Bonferroni correction is below the diagonal.

* p<0.05.

** p<0.01.

NS = not significant.

## Discussion

Young of the year winter flounder living in New York estuaries exhibit relatively high genetic diversity in terms of microsatellite allelic richness, yet the low m-ratios observed suggest that rare alleles may have been lost within individual sample locations. Genetic diversity was weakly geographically partitioned between some of the bays, with distance between sites playing a small but significant role in driving this structure. All bays were out of HWE due to large excesses of homozygotes across 10 of 11 loci, leading to high inbreeding coefficients (F_IS_) in all bays. Many individuals also exhibited very high internal relatedness and individuals' genotypes exhibited a high proportion of homozygous loci. These patterns could not be explained by an artificial inflation of homozygosity resulting from technical issues. Large allelic dropout produces a pattern skewed towards homozygotes for small alleles, which did not occur in the LI winter flounder. If null alleles were at high enough frequencies at all loci to drive these patterns, we should have observed many null homozygotes (blanks for certain loci) when we had none. We would probably have also been able to amplify null alleles in homozygotes by lowering temperatures, but this did not occur. Inadvertent sampling of closely related individuals can also generate HWE deviations of this nature, but few (∼6%) of the sampled young-of-the-year fish exhibited high relatedness (r>0.25) ruling out a Wahlund effect due to sampling of closely related individuals. A Wahlund effect due to undetected population structure is also unlikely, as no cryptic genetic structure within any of the bays was detected using Structure.

The most likely explanation for these large deviations in HWE is that inbreeding is occurring in LI winter flounder. Similar evidence for inbreeding has been documented in a wide variety of terrestrial and freshwater animals (e.g. wolves (*Canis lupus*) [57], deer (*Cervus elaphus*) [58], wasps (*Ancistrocerus antilope*) [59], brook trout (*Salvelinus fontinalis*) [60]), but relatively few marine fish [17], [61]. A number of other studies have found that marine fish populations can exhibit strong deviations from HWE (e.g., European anchovy (*Engraulis encrasicolus*) [15], whitefish (*Coregonus lavaretus lavaretus*) [20], rockfish (*Sebastes melanops*) [21]), including other flatfish and winter flounder in other regions [24], [36], [62]–[65]. In these studies excess homozygosity is generally attributed to technical or sampling artifacts and alternative biological explanations, such as inbreeding, are not explicitly tested. Our results and those of Hoarau et al. [17] suggest that inbreeding should be routinely considered as a potential cause of HWE deviation in heavily exploited marine fish. Loci with the largest deviations from equilibrium expectations are frequently discarded in studies of wild animal populations based on the assumption that they have null alleles [66]. While this is always a possibility, it is important to consider that biological explanations are also an option, especially if the deviations are chronic at multiple loci. This will enable rigorous testing of these alternative explanations and a less biased assessment of the magnitude and causes of HWE deviation in marine fish.

We propose the inbreeding observed in LI populations may be due a confluence of a small spawning population in each bay and a propensity of these fish to spawn in their natal estuary. It has generally been assumed that marine fish exhibit panmictic population structure and do not require management at the sub-population level [10]; however studies have shown that population structure is important in many species [9]. Previous research on LI proposes the existence of multiple distinct behavioral groups with observations indicating the presence of resident and migratory individuals termed “bay fish” and “offshore fish”, respectively [27], [29], [67]. Poole [67] estimated morphometric differences and variation in age and growth across four south shore bays of LI. It is not yet clear if these contingents are genetically differentiated [9], [29], but if they are, then including individuals from both groups in the same analysis could cause heterozygote deficiencies relative to HWE expectations. However, this hypothesis is unlikely to explain the strong HWE deviations we observed. Bayesian clustering failed to detect any strongly differentiated groups that could correspond to “bay” and “offshore” contingents within bays and none of the young-of-the-year fish from different cohorts in the same year sampled in a given bay were genetically differentiated from one another. Although we report small but significant F_ST_ along the south shore of LI and Peconic Bays and a weak pattern of IBD, these estimates of genetic differentiation are confounded by inbreeding at the individual level within the bays we sampled. Since there were significant differences in IR between bays we cannot assume that this bias is the same for each bay. More direct methods for assessing migration rates between bays (tagging, telemetry or otolith microchemistry) are needed to assess genetic differentiation between winter flounder in these bays.

We suggest that relatively few mature adults are contributing to each generation, resulting in a high proportion of related fish spawning with each other. Like any broadcast spawning species it is probable that there is large variation in reproductive success in this species due to high larval and pre-recruit mortality [35]. When spawning populations are large, inbreeding is unlikely even despite these characteristics. All recent indicators, however, show that spawning populations have reached extremely low levels in New York estuaries [29], [68], [69]. We have no direct evidence that spawning fish are related because sampled individuals were YOY rather than spawning adults. However, our estimates of effective number of breeders producing the cohorts we sampled were consistent with the premise that there are relatively few spawning adults in these bays, because all estimates were in the tens to hundreds of individuals. It is important to note that these estimates do not necessarily reflect the spawning population for the entire bay. They may only be representative of the parts of each bay that we sampled since bays were not sampled randomly and flounder are patchily distributed [29].

Inbreeding could directly contribute to the failure of some marine fish to recover from exploitation as it has been linked to lower survival and reproduction rates and lower resistance to disease and environmental stress [70] and can have a significant effect on extinction risk [71], with persistence time of inbred endangered species being reduced 17.5–28.5% [72]. Additionally, the effect of inbreeding depression is multiplied in a stressful environment [73]. A recent study by Bickley et al. [74] tested the effect of an endocrine disrupter (the fungizide clotrimazole) on reproduction on a model fish (zebrafish, *Danio rerio*) in a laboratory setting. They confirmed that inbreeding has a much stronger effect when combined with the exposure to a chemical stressor, resulting in lower embryo viability and few offspring. Western populations of winter flounder on LI, particularly those in Jamaica Bay, are exposed to anthropogenic habitat degradation, particularly large amounts of municipal sewage effluent which has been shown to contain estrogenic compounds [75], [76]. Additionally there is evidence that winter flounder from Jamaica Bay demonstrate signs of endocrine disruption linked to the estrogenic compounds found there [77]. This environmental degradation of LI bays combined with inbreeding depression may contribute to the ongoing recruitment failure of winter flounder and should be further investigated.

Marine fish have historically been managed without an underlying concern that populations could be reduced to the point where they would become vulnerable to local extinction and processes that reduce their genetic diversity. We show that the effective number of breeders is so low in some parts of the winter flounder range that it is able to be estimated using genetic approaches that work most effectively for small populations. Although we observe only weak evidence of a genetic bottleneck at this stage, the number of spawning adults is sufficiently low for inbreeding to be occurring. Given similar findings in genetic studies of North Sea plaice [17] and evidence of local population structure in other species, we suggest that resource managers should consider the possibility that exploited marine fish are vulnerable to local extinction and inbreeding.
